# 

**Published:** 1889-11-16

**Authors:** 


SATURDAY, NOVEMBER 16th, 1889.

**Charitable Appeals and Charities** - 97

**Words of Consolation**—Prayer—Joy - 98

**Invalid Children's Aid Association** - 99

**A Home to Die In** - 100

**The Hospital Workshop.** XV.—The Birmingham District Nursing Society - 101

**Help for the London Poor** - 101

**Everybody's Page** - 103

**Notes and News** - 104

**Annotations.**—A City of Cesspools—Sir Jas. Whitehead, Bart. 106

**The Practitioner's Outlook and Retrospect:**

MEDICINE—Phthisis—Alcohol in Medicine—Albuminuria in Pregnacy—Pernicious Anæmia - 107

SURGERY—Hernia Cerebri—Trephining for Epilepsy - 108

THERAPEUTICS—The Ice Treatment—Atropine—Treatment of Yellow Fever - 109

**An Asylum Infirmary for 100 Beds.** With full plans - 111

**Scraps and Gleanings** - 112

### EXTRA SUPPLEMENT—THE NURSING MIRROR.

**En Passant.**—Registration of Midwives—Exhibition of Nursing Uniforms—A New Cap—Short Items—Warwick House, Leeds—Then and Now—Notes from Oxford - xxix
**The Management of Children.**—II. Diseases of Infancy xxx
**Examination Questions.**—Prize Answer for October - xxx
**Everybody's Opinion.**—Ladies as District Nurses—District Nursing—The Scandal at Hope Infirmary—Asylum Attendants—Infantile Mortality - xxxi
**Presentations—Notices—Notes and Queries** - xxxii
**Appointments—Vacancies—Amusements** - xxxii

